# Recurrent symptomatic urolithiasis in a patient with cystic fibrosis

**DOI:** 10.1007/s00467-024-06433-2

**Published:** 2024-06-25

**Authors:** Sibel Yel, Ismail Dursun, Mehmet Köse, Aslıhan Kiraz, Muammer Hakan Poyrazoglu, Munis Dündar

**Affiliations:** 1 Department of Pediatric Nephrology, Erciyes University Medical Faculty, Kayseri, Turkey; 2 Department of Pediatric Pulmonology, Erciyes University Medical Faculty, Kayseri, Turkey; 3 Department of Medical Genetics, Erciyes University Medical Faculty, Kayseri, Turkey

**Keywords:** Children, Cystic fibrosis, Urolithiasis, Hyperoxaluria, Lumasiran

## Abstract

A 6-month-old girl, previously diagnosed with cystic fibrosis (CF), was admitted to hospital for nephrolithiasis. Her parents were first-degree cousins. The patient underwent endoscopic stone management. Despite no family history of stones and medical treatment with potassium citrate, the patient developed recurrent renal stones and atypical urinary tract infections during follow-up. Basic investigations were all normal. Due to consanguinity and early presentation of nephrolithiasis, metabolic causes such as cystinuria and hyperoxaluria were considered. Cystinuria was excluded due to normal cystine levels. High urinary oxalate excretion was found as expected due to absorptive (secondary) hyperoxaluria in CF patients. An early stone burden in the patient with a history of medical treatment and consanguinity led us to perform a genetic testing. Genetic testing revealed a missense homozygous variant in exon 1 of the *AGXT* gene. The patient was diagnosed with primary hyperoxaluria type 1. Two rare life-threatening genetic diseases were found together in the same child.

## Case presentation

A 6-month-old girl, previously diagnosed with cystic fibrosis (CF), presented to the emergency department with fever and restlessness. She had microscopic hematuria, pyuria; and elevated acute phase reactants. Because her abdominal ultrasound showed nephrolithiasis, she was referred to our pediatric nephrology unit for further evaluation.

Genetic testing previously revealed that she had a homozygous pathogenic mutation on the cystic fibrosis transmembrane conductance regulator (*CFTR*) gene (c.1521_1523 del p.Phe508del). The *CFTR* gene c.1521_1523 del p.Phe508del (NM_000492.4) variant was classified as pathogenic according to the ACMG (American College of Medical Genetics and Genomics) criteria (PS4, PS1, PM4, PM2) during the variant interpretation (PMID: 25741868).

Her parents were first-degree cousins and the patient was an in vitro fertilization term baby. Her weight was 6.5 kg (10th percentile) and her length was 64 cm (3–10th percentile). Kidney function tests and electrolytes were within normal range. Abdominal computed tomography revealed a right distal ureteral stone of 8 mm without hydronephrosis and bilateral renal parenchymal stones of 2–3 mm. The patient underwent endoscopic stone treatment with ureteral stenting. Baseline investigations including serum bicarbonate, calcium (Ca), parathormone (PTH), and urinary Ca excretion were all unremarkable. Abdominal computed tomography showed no additional kidney abnormalities.

Despite no family history of stones and medical treatment with potassium citrate, the patient developed recurrent renal stones and urinary tract infections with atypical microorganisms. Further investigations were performed for differential diagnosis. To exclude vitamin D hypervitaminosis, the serum 25-OH vitamin D level was tested and found to be within the normal range. Voiding cystouretrography was not considered. Infections were associated with urolithiasis.

Genetic and metabolic causes such as cystinuria and hyperoxaluria were identified. Urinary cystine levels were within the normal range. The spot urinary oxalate-to-creatinine ratio was 0.28 mg/mg, which was considered very high in relation to our laboratory reference values (< 0.20 mg/mg) for her age. Absorptive (secondary) hyperoxaluria seemed to be the causative factor of the patient’s stone disease. Despite medical treatment, the patient’s early stone burden and consanguinity history led us to perform genetic testing.

A missense homozygous pathogenic variant in exon 1 of the *AGXT* gene (c.121G > A p.Gly41Arg) was detected. The *AGXT* gene c.121G > A p.Gly41Arg (NM_000030.3) variant was classified as pathogenic according to the ACMG criteria (PM3, PS3, PS1, PM5, PM1, PP2, PM2, PP3) during variant interpretation (PMID: 25741868). The patient was diagnosed with primary hyperoxaluria type 1.

In addition to potassium citrate treatment and general hydration advice, pyridoxine treatment was started. The patient is now being prepared for treatment with lumasiran.

## Discussion

Nephrolithiasis has a multifactorial etiology. The prevalence of monogenic nephrolithiasis in children is reported to be up to 30% [1]. Identification of a monogenic cause of nephrolithiasis facilitates optimal management. However, an optimal management strategy may also prevent chronic kidney disease. The incidence of nephrolithiasis is increased in patients with CF, although the prevalence of symptomatic urolithiasis is low. Hyperoxaluria is one of the predisposing risk factors for the increased incidence. A recent study reported hypercalciuria, hyperuricosuria, and hyperoxaluria in children with CF [2].

Cystic fibrosis is an autosomal recessive, life-shortening disease caused by mutations in the cystic fibrosis transmembrane conductance regulator (*CFTR*) gene. Oxalate secretion in the intestine is mediated by *SLC26A6* which contributes to homeostasis of oxalate. The interaction of *CFTR* with *SLC26A6* stimulates its transport activity as a Cl-base exchanger. Genetic deletion of the *CFTR* gene causes a major defect in intestinal oxalate secretion due to reduced *SLC26A6* expression [3]. Patients with CF have an increased risk of hyperoxaluria and calcium oxalate nephrolithiasis due to absorptive hyperoxaluria [4].

Primary hyperoxaluria is a distinct genetic entity associated with excessive oxalate production and urinary excretion resulting in systemic oxalate deposition, and kidney failure. Primary hyperoxaluria type 1 (PH1) is the most severe form of the disease. It is caused by mutations in the liver-specific alanine glyoxylate and serine pyruvate aminotransferases [5]*.* In 20–50% of cases, severe kidney failure occurs before diagnosis. Nephrocalcinosis is an independent risk factor for kidney failure. Traditional treatments such as hyperhydration, citrate, and low oxalate diet are symptomatic interventions. These therapeutic options are inadequate, and the new future therapeutic strategy options for PH1 are mainly targeted therapies,

Lumasiran is a new RNAi therapy. It inhibits oxalate synthesis by silencing the expression of the gene encoding glycolate oxidase. As a result, it reduces oxalate levels in urine and plasma, offering new hope for the treatment of PH1 [5].

## Conclusion

Recurrent nephrolithiasis in infancy requires careful management. A multifactorial etiology including metabolic and genetic factors should be investigated. Early presentation of urolithiasis, especially small bilateral stones, rather than recurrence of calculous disease, makes a metabolic cause more likely.

A small stone in a baby, especially if there is consanguinity in the family history, may be the first clue of a life-threatening treatable genetic disease.

## Summary

### What is new?

Two rare life-threatening genetic diseases were identified together in the same baby. A known etiology for each disease presentation is not always the only true cause. A follow-up strategy of suspicion is needed.
